# Functional analysis of thyroid hormone receptor beta in *Xenopus tropicalis* founders using CRISPR-Cas

**DOI:** 10.1242/bio.030338

**Published:** 2018-01-15

**Authors:** Yuto Sakane, Midori Iida, Takashi Hasebe, Satoshi Fujii, Daniel R. Buchholz, Atsuko Ishizuya-Oka, Takashi Yamamoto, Ken-ichi T. Suzuki

**Affiliations:** 1 Department of Mathematical and Life Sciences, Hiroshima University, Higashi-Hiroshima, Hiroshima 739-8526, Japan; 2 Department of Bioscience and Bioinformatics, Kyushu Institute of Technology, Iizuka, Fukuoka 820-8502, Japan; 3 Department of Biology, Nippon Medical School, Musashino, Tokyo 180-0023, Japan; 4 Department of Biological Sciences, University of Cincinnati, 312 Clifton Ct., Cincinnati, OH, 45221, USA

**Keywords:** Metamorphosis, Thyroid hormone receptor, CRISPR-Cas, *Xenopus tropicalis*

## Abstract

Amphibians provide an ideal model to study the actions of thyroid hormone (TH) in animal development because TH signaling via two TH receptors, TRα and TRβ, is indispensable for amphibian metamorphosis. However, specific roles for the TRβ isoform in metamorphosis are poorly understood. To address this issue, we generated *trβ-*disrupted *Xenopus tropicalis* tadpoles using the CRISPR-Cas system*.* We first established a highly efficient and rapid workflow for gene disruption in the founder generation (F0) by injecting sgRNA and Cas9 ribonucleoprotein. Most embryos showed severe mutant phenotypes carrying high somatic mutation rates. Utilizing this founder analysis system, we examined the role of *trβ* in metamorphosis. *trβ*-disrupted pre-metamorphic tadpoles exhibited mixed responsiveness to exogenous TH. Specifically, gill resorption and activation of several TH-response genes, including *trβ* itself and two protease genes, were impaired. However, hind limb outgrowth and induction of the TH-response genes, *klf9* and *fra-2*, were not affected by loss of *trβ*. Surprisingly, *trβ-*disrupted tadpoles were able to undergo spontaneous metamorphosis normally, except for a slight delay in tail resorption. These results indicate TRβ is not required but contributes to the timing of resorptive events of metamorphosis.

## Abstract (precis)

**Summary:** Using knocked-out *Xenopus tropicalis* founders generated by CRISPR-Cas, TRβ was shown to have significant effects on developmental timing but is not required for completion of metamorphosis.

## INTRODUCTION

Thyroid hormone (TH), acting through TH receptors (TRs), plays critical roles in various biological processes, including development, growth, and metabolism (Yen, 2001; Cheng et al., 2010; Waung et al., 2012; Brent, 2012; Ortiga-Carvalho et al., 2014; Mullur et al., 2014). TRs belong to the nuclear receptor superfamily, and two types of TRs, TR alpha (TRα) and TR beta (TRβ), are coded in separate gene loci in vertebrates (Wahli and Martinez, 1991; Tsai and O'Malley, 1994). TRs are ligand-dependent transcription factors that heterodimerize with the retinoid X receptor (RXR), and the heterodimers bind to thyroid hormone response elements (TREs) in the promoter or enhancer of TH-response genes to regulate their expression (Perlmann et al., 1993; Wu et al., 2001). TRs recruit co-repressors in the absence of TH and co-activators in the presence of TH to TREs to repress and activate target gene expression, respectively, as described by the ‘dual function model’ for the role of TRs during development (Sachs and Shi, 2000; Shi, 2009; Grimaldi et al., 2013).

Amphibian metamorphosis involves unique and dynamic morphological and physiological changes from larva to adult: resorption of gills and tail; remodeling of skin and intestinal tissues; and growth of the limbs (Tata, 1999; Furlow and Neff, 2006; Brown and Cai, 2007). The functions and mechanisms of action of TH and TRs in metamorphosis have been well-studied in various amphibians over several decades. In particular, *Xenopus laevis* and *Xenopus tropicalis* provide excellent models for TH-dependent metamorphosis (Buchholz, 2017). Two TR coding genes, *trα* and *trβ*, are present in the *Xenopus* genome, as in other vertebrates (Yaoita and Brown, 1990). *trα* is widely expressed before metamorphosis, and the level of its expression is maintained until the end of metamorphosis (Yaoita and Brown, 1990; Wang et al., 2008). In contrast, *trβ* mRNA is also widely expressed but at much lower levels during pre- and pro-metamorphosis (Yaoita and Brown, 1990; Wang et al., 2008). After TH secretion by the thyroid gland commences (stage 54) (Nieuwkoop and Faber, 1994; Leloup and Buscaglia, 1977), *trβ* expression levels increase strongly as the TH concentration rises in the plasma (Yaoita and Brown, 1990) until both reach a peak at the climax of metamorphosis (stage 61–62) and then TH and *trβ* levels attenuate toward the end of the metamorphosis. The expression of *trβ* follows the plasma profile of TH due to TREs in its promoter allowing for self-induction in the presence of TH (Tata, 1993; Ranjan et al., 1994; Wang et al., 2008).

Genome-editing techniques have greatly improved the exploration of the roles of TRs in metamorphosis (Choi et al., 2015, 2017; Wen and Shi, 2015, Wen et al., 2017) beyond what was possible using overexpression of dominant negative mutant TRs in transgenic animals (Schreiber et al., 2001; Buchholz et al., 2003). Using TALEN knock-out techniques in *X. tropicali*s, we and others have demonstrated that *trα* regulates the timing of amphibian metamorphosis (Choi et al., 2015; Wen and Shi, 2015; Choi et al., 2017; Wen et al., 2017). In general, TRα knock-out phenotypes were mild, such that homozygous TRα knock-out animals have apparently normal survival and fertility. However, due to its dramatic self-regulation, TRβ is thought to be the master regulator at the top of a gene regulation cascade required for metamorphosis, and therefore is expected to play a crucial role in driving radical changes at the climax stage of frog metamorphosis. However, the details of its involvement and degree of functional overlap with TRα during metamorphosis are poorly understood.

The CRISPR-Cas system has several advantages for loss-of-function analysis compared with other genome-editing tools used for *X. tropicalis*, including high efficiency, convenience, and cost-effectiveness (Blitz et al., 2013; Nakayama et al., 2013; Shigeta et al., 2016; Naert et al., 2016). Thus, in this study, we first established a workflow to generate mosaic knock-out founders (crispants) carrying high rates of somatic mutations using injection of the sgRNA/Cas9 ribonucleoprotein (RNP). Next, we generated *trβ* crispants to examine the biological significance of *trβ* in amphibian metamorphosis. In agreement with previous pharmacological studies (Furlow et al., 2004; Denver et al., 2009), exogenous TH did not induce gill resorption in *trβ* crispants at the pre-metamorphic stage. Gene expression analysis indicated that some but not all TH-response genes lacked induction by TH in the crispants. Surprisingly, *trβ* crispants completed metamorphosis, though after a delay. Our results suggest that TRβ, like TR*α*, is not required to complete metamorphosis but has metamorphic roles distinct from those previously determined for TRα.

## RESULTS

### sgRNA/Cas9 ribonucleoprotein effectively produces knocked-out founders in *Xenopus tropicalis*

In this study, we employed Cas9 protein instead of Cas9 mRNA for injection, to maximize gene disruption efficiency, and we established an effective and convenient protocol for analysis of loss-of-function in *X. tropicalis* founders (Fig. S1). As proof of concept, we targeted the melanin synthesis-related genes for *tyrosinase* (*tyr*) and *oculocutaneous albinism 2* (*oca2*) as model genes. *tyr* sgRNA was designed within the exon region, whereas *oca2* sgRNA was designed across the exon-intron junction (Fig. S2A). Each sgRNA was synthesized by T7 RNA polymerase-based *in vitro* transcription using PCR templates. sgRNA and Cas9 protein were incubated *in vitro* to form the RNP complex in 150 mM KCl and 20 mM HEPES, according to previous reports (Burger et al., 2016). The RNPs were then injected into *X. tropicalis* one-cell-stage fertilized eggs. As expected, injection of *tyr* or *oca2* sgRNA/Cas9 RNP caused albinism phenotypes in almost all cases (Fig. S2B). About 70% of these crispants showed severe complete loss of pigmentation, while the proportion with developmental defects was comparable to uninjected controls, about 15-30% (Fig. S2C). Surprisingly, amplicon sequencing analysis of these on-target sites revealed that somatic mutation rates reached up to 100% in all crispants analyzed, where no wild-type allele reads could be identified (Fig. S2D,E). According to our amplicon sequencing data, we actually detected only 5 to 15 types of mutant alleles and no wild-type allele per animal in *tyr* and *oca2* crispants exhibiting severe albino phenotypes, which suggests that mutagenesis by Cas9 RNP occurred at a very early developmental stage in these animals. Therefore, it seems that the 2000 reads were saturated, suggesting that most if not all cells of an individual were mutant. Frameshift mutation rates were 89% in *tyr* crispants which is well above the expected 66%, likely because individuals with severe mutations were chosen for sequencing. These results demonstrate that this Cas9 RNP-based protocol enables production of mosaic knocked-out founders (crispants; Burger et al., 2016) with high frequency and low toxicity.

### Generation of *trβ* crispants in *X. tropicalis*

**Fig. 1.**
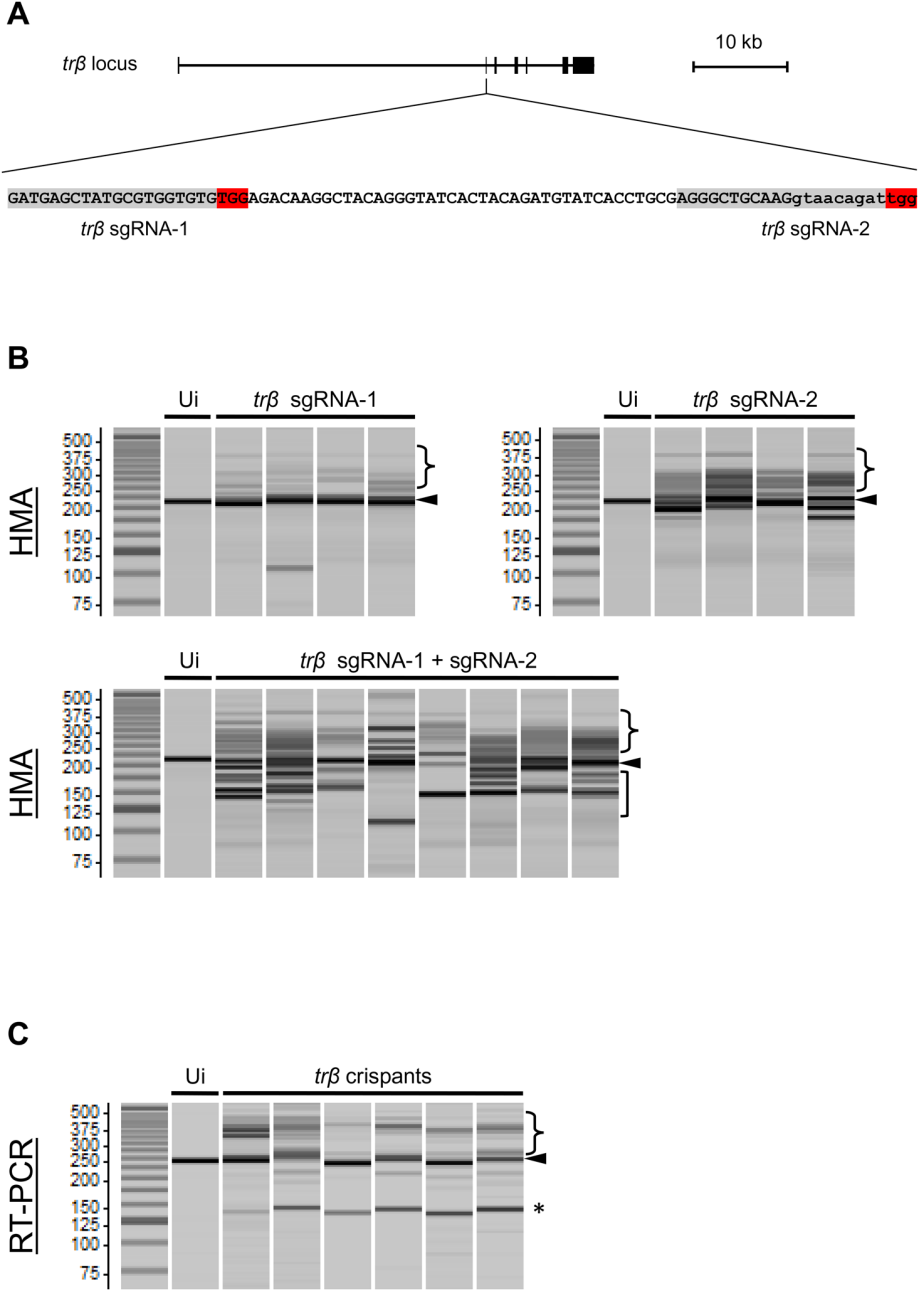
***trβ* sgRNA/Cas9 RNP leads to *trβ* gene disruption in *X. tropicalis* founders.** (A) Schematic illustration of the sgRNA targeting sequences on the 2nd exon and 2nd intron of the *trβ* gene in *X. tropicalis*. One of the sgRNAs was designed in the coding region (*trβ* sgRNA-1) and the other was designed across the exon-intron junction (*trβ* sgRNA-2). Highlights in red and gray denote the protospacer adjacent motif (PAM) and the 20 bp target sequences of sgRNA, respectively. Exon and intron sequences are indicated by capital and small letters, respectively. (B) Genotyping by HMA in uninjected and injected embryos. PCR products encompassing sgRNA target sites were analyzed using a microchip electrophoresis system. Heteroduplex bands and multiple short bands are shown in the sgRNA/Cas9 RNP-injected embryos (crispants). Arrowheads, wild-type original bands; curly brackets, up-shifted heteroduplex bands; square brackets, deleted bands. (C) RT-PCR was performed using total RNA from stage 61/62 tadpoles with primers designed upstream and downstream of the 2nd exon. In addition to the original band (arrowhead), about 101 bp deleted bands were detected in crispants caused by skipping of the 2nd exon (asterisk) in *trβ* crispants. Heteroduplex bands were also detected in crispants (brackets). Each lane represents an individual animal. Ui, uninjected control.

**Fig. 2.**
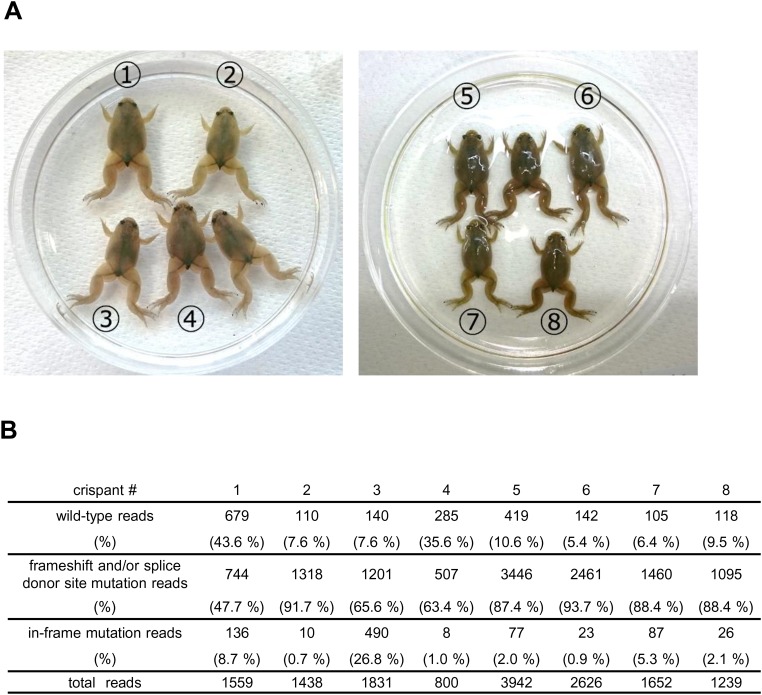
**Amplicon sequencing analysis of on-target sites in *trβ* crispants.** (A) Image of *trβ* crispants at stage 66. Genomic DNA was extracted from whole bodies of eight *trβ* crispants and the sgRNA target region was PCR-amplified using barcoded primers. PCR amplicons were subjected to amplicon sequencing according to Materials and Methods. Numbers indicate each crispant that was analyzed by amplicon sequencing in B. (B) The results of wild-type and mutant allele reads in each *trβ* crispant. All sequenced reads were classified into three groups; wild-type reads, frameshift and/or splice donor site mutation reads, in-frame mutation reads.

To investigate the function of TRβ in *X. tropicalis* metamorphosis, we disrupted the *trβ* gene using this CRISPR-based system to generate mutant founders. Two sgRNAs were designed in the coding region of the 2nd exon and across the 2nd exon-2nd intron junction to produce frameshift mutations and splicing errors, respectively (Fig. 1A). We injected Cas9 RNP with each of the two sgRNAs, or with mixtures of the two, into one-cell-stage fertilized eggs. On the following day, normally developed embryos were collected and a sub-sample selected for genotyping to examine the efficiency of disruption at the on-target sites. In a heteroduplex mobility assay (HMA), heteroduplex formation was detected in all *trβ* crispants, indicating that disruption of the target sites occurred in the *trβ* gene (Fig. 1B). As expected, multiple shorter PCR bands were observed in all embryos injected with the two sgRNAs/Cas9, consistent with these bands being small deletions between the two sgRNA-targeting sites. Injection of both sgRNA/Cas9 RNPs probably induced frameshift mutations and/or small deletions at the exon-intron boundary causing splicing errors (Radev et al., 2015). To examine the possible splicing errors in more detail, we analyzed *trβ* mRNA from whole bodies of stage 61/62 crispants by RT-PCR using primers designed in the 1st and 3rd exons. In *trβ* crispants injected with both sgRNAs, 2nd exon deleted bands (101 bp shorter) as well as other non-wild-type bands were present, indicating numerous perturbations of *trβ* mRNA in the metamorphic climax stage tadpoles (Fig. 1C). We sequenced eight metamorphosed crispants, which showed high somatic mutation rates (48-94%) (Fig. 2).

### Limb development, but not gill resorption, is induced by T3 in pre-metamorphic *trβ* crispants

**Fig. 3.**
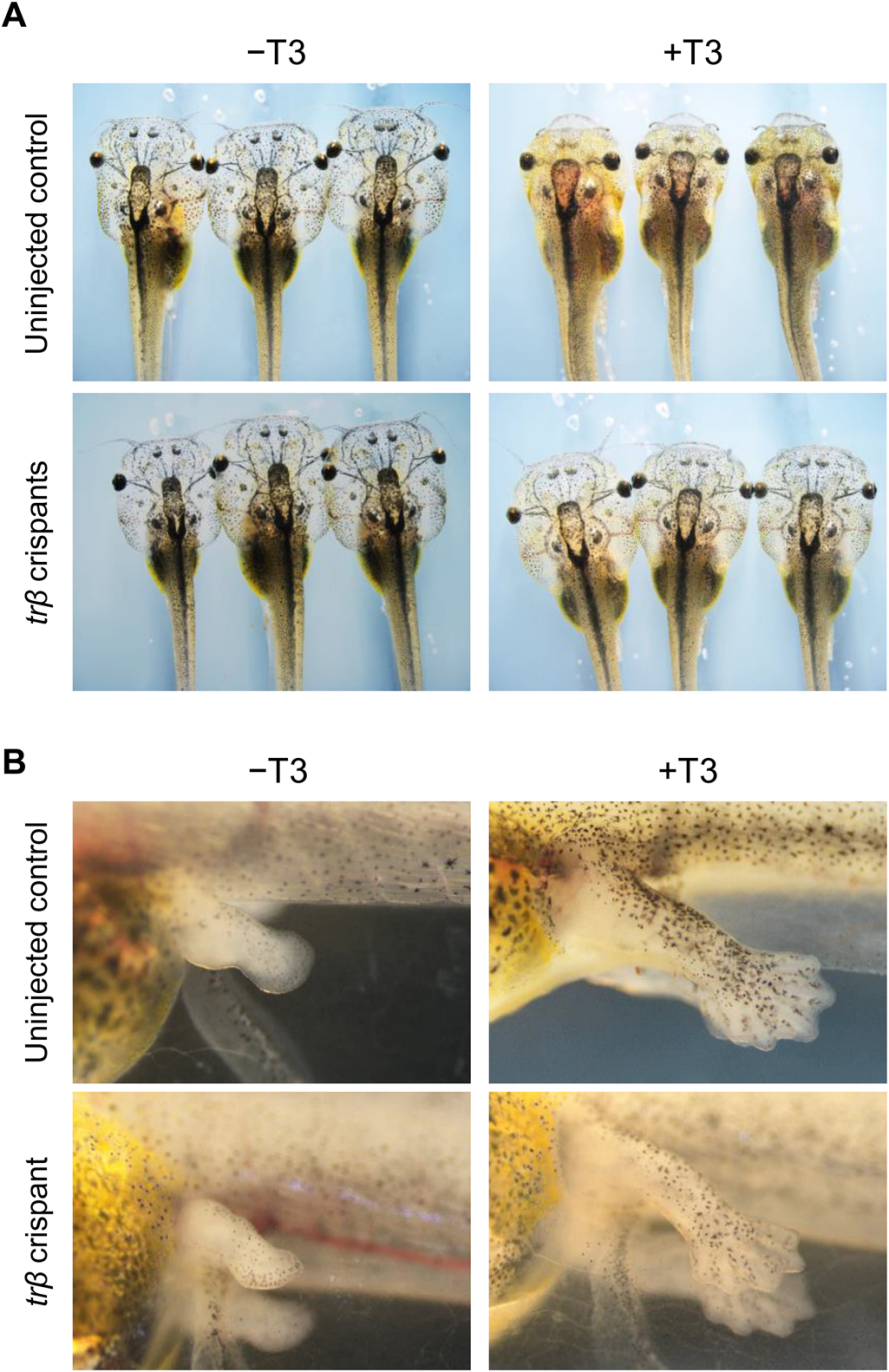
**Morphological changes induced by T3 treatment in *trβ* crispants.** (A) Uninjected control tadpoles and *trβ* crispants at pre-metamorphic stage (stage 52–54) were treated with or without 10 nM T3 for 3 days and morphological changes observed. After 3 days of T3 treatment, regression of gills was observed in all uninjected control tadpoles (*n*=44/44), whereas these morphological changes were not induced in most of the *trβ* crispants (*n*=51/54). In contrast, morphological change did not occur in the gills of both uninjected control tadpoles and *trβ* crispants without T3 treatment. (B) Hind limb development was induced by T3 treatment in all uninjected control tadpoles (*n*=44/44) and *trβ* crispants (*n*=54/54). *N* values represent the total numbers of tadpoles in three independent experiments.

No differences in growth and development were observed in embryogenesis and pre-metamorphic development in *trβ* crispants versus controls, as expected because the expression level of *trβ* is very low during embryonic and pre-metamorphic stages (Yaoita and Brown, 1990; Wang et al., 2008). To investigate responsiveness to exogenous TH in pre-metamorphic *trβ* crispants, we treated stage 52–54 tadpoles with or without 10 nM 3,3,5-triiodo-L-thyronine (T3) for 3 days and examined external morphology. As expected, all uninjected control tadpoles responded to the exogenous T3 and exhibited characteristic gill resorption (*n*=44/44) (Fig. 3A). In contrast, almost all of the *trβ* crispants were resistant to T3 treatment and showed no, or slight, morphological changes in the gills (*n*=51/54). Gill resorption equivalent to that in the controls was observed in only 5.5% of crispants, which may represent unintentionally uninjected embryos (*n*=3/54). However, hind limbs responded to T3 and precociously developed in both uninjected controls and *trβ* crispants (Fig. 3B). In the absence of T3, gill and limb morphology were unchanged in both groups of tadpoles. These results suggest that the gills exhibit high responsiveness to TH via TRβ-mediated signaling during T3-induced metamorphosis, but the hind limbs do not.

### Impaired induction of TH-response genes in *trβ* crispants

**Fig. 4.**
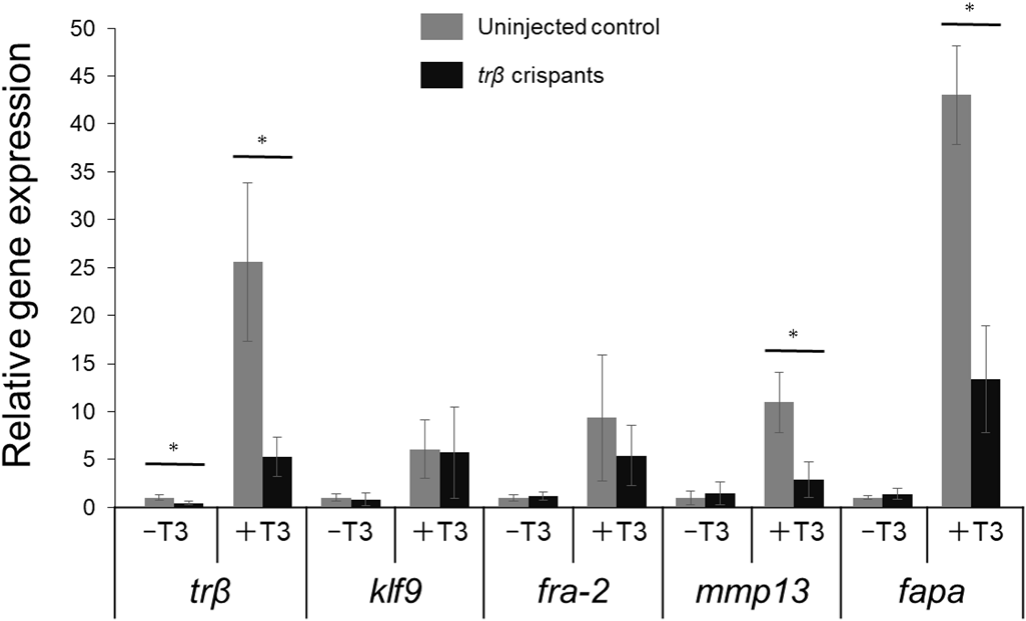
**Impaired induction of TH-response genes in *trβ* crispants.** mRNA expression of five TH response genes, *thyroid hormone receptor β* (*trβ*), *krüppel-like factor 9* (*klf9*), *fos-related antigen-2* (*fra-2*), *matrix metallopeptidase 13* (*mmp13*), and *fibroblast activation protein alpha* (*fapa*), was analyzed by qPCR. Uninjected control tadpoles and *trβ* crispants at pre-metamorphic stage (stage 52–54) were treated with or without 10 nM T3 for 3 days and at least four tadpoles were pooled per sample (*n*=3) in three independent experiments. The expression levels were normalized by *rpl8*. Expression changes by T3 induction were calculated relative to those of control animals without T3 treatment. Error bars represent ±s.d. (*n*=9). The three independent experiments exhibited similar trends. Asterisks indicate significant differences between the uninjected control and *trβ* crispants: *P<0.005; Welch's *t*-test.

Next, we extracted total RNA from whole bodies of T3-treated and untreated tadpoles and analyzed the induction of the well-known TH-response genes, *trβ*, *krüppel-like factor 9* (*klf9*), *fos-related antigen-2* (*fra-2*), *matrix metallopeptidase 13* (*mmp13*), and *fibroblast activation protein alpha* (*fapa*) using quantitative RT-PCR. Surprisingly, not all direct TH response genes responded in the same way to loss of *trβ* (Fig. 4). Two of the direct response genes, key transcription factors related to metamorphosis, *klf9* and *fra-2*, were normally induced by T3 in both uninjected control and *trβ* crispants. However, mRNA levels of *trβ* itself, which is autoregulated by TH, and the two protease genes, *mmp13* and *fapa*, were significantly lower in *trβ* crispants than in control tadpoles. Therefore, some but not all TH direct response genes require TRβ for activation. The basal expression level of *trβ* mRNA in the crispants was significantly lower than that of the uninjected controls in the absence of T3 (Fig. 4; Fig. S3), possibly due to disruption of *trβ* function mediated by splicing error and nonsense-mediated mRNA decay (NMD; Popp and Maquat, 2016).

### *trβ* crispants accomplish normal metamorphosis, although slightly delayed

**Fig. 5.**
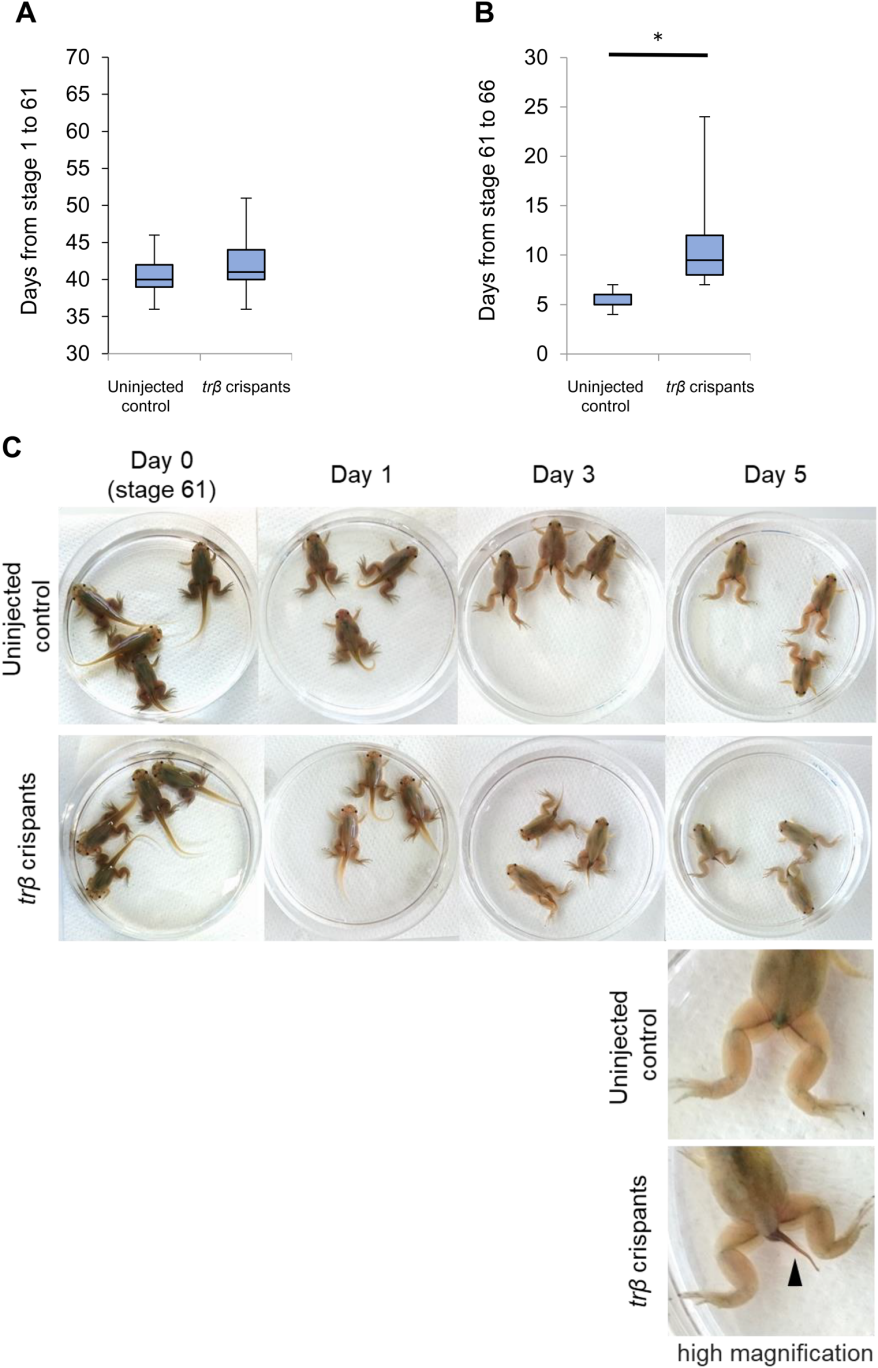
**Delay of tail resorption in *trβ* crispants during natural metamorphosis.** (A) The days from fertilization to stage 61 are indicated in a box plot for uninjected control tadpoles (*n=*46) and *trβ* crispants (*n*=39). There were no significant differences. (B) The days from stage 61 to stage 66 are indicated in a box plot for uninjected control tadpoles (*n*=45) and *trβ* crispants (*n*=38). *trβ* crispants required significantly more days to reach stage 66 (Welch's *t*-test, *P<0.05). *N* values represent the merged total numbers of tadpoles from two independent experiments. Box and whiskers show the interquartile range and maximum and minimum data points, respectively. (C) Time course of tail resorption in uninjected control tadpoles and *trβ* crispants. Uninjected control tadpoles and *trβ* crispants of the same age were selected from the same clutch at stage 61 and gill resorption observed at 1 days, 3 days, and 5 days after stage 61 (day 0). Uninjected control tadpoles completed tail resorption by day 5. In contrast, the tail remained in *trβ* crispants on day 5 (an arrowhead in the high magnification image) and a longer time was required to reach the end of metamorphosis.

**Fig. 6.**
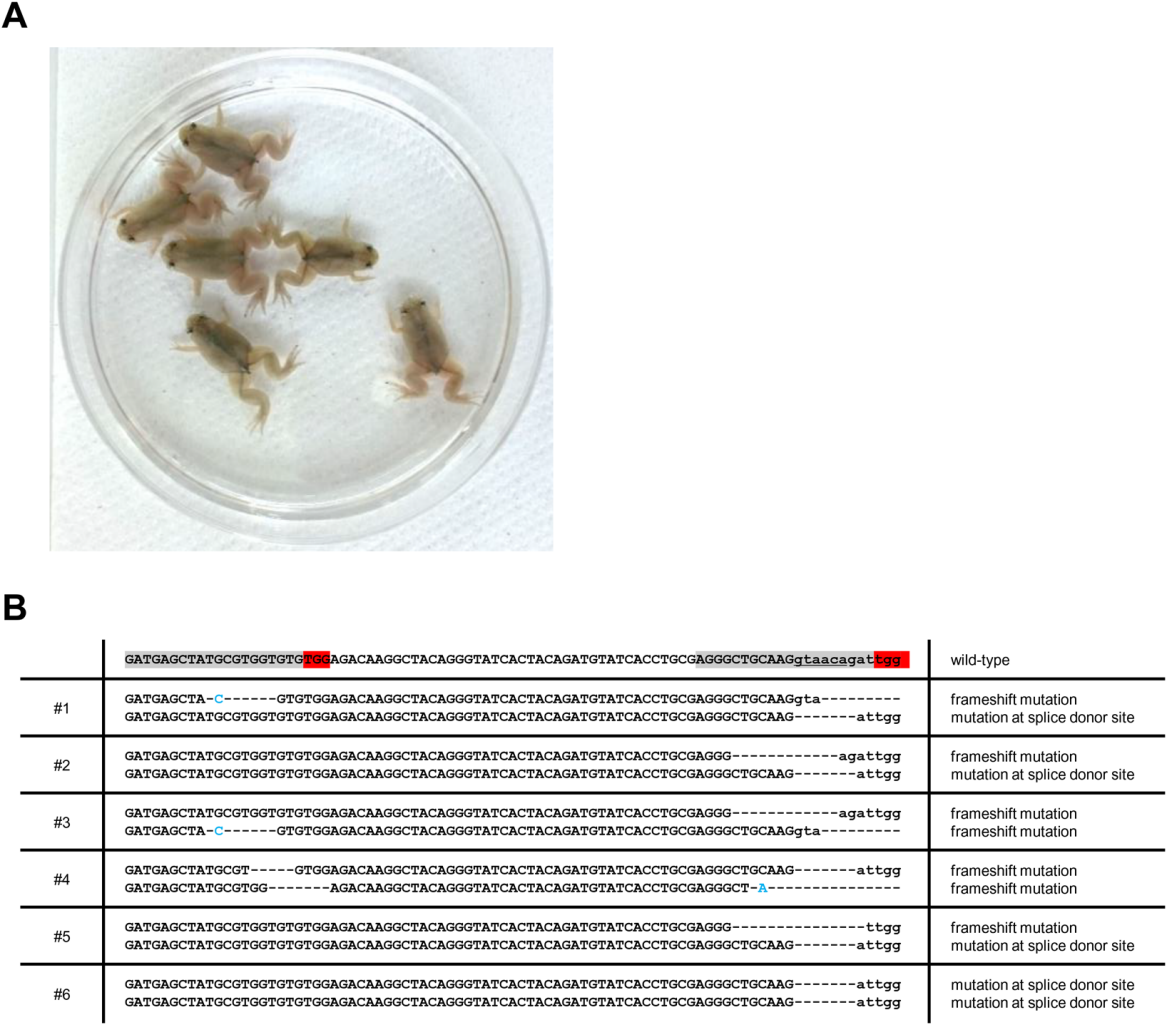
**F1 *trβ* mutants accomplish natural metamorphosis.** (A) Image of F1 offspring produced by mating of the F0 crispants. (B) Mutation sequences from each F1 offspring. PCR products of *trβ* target site from six F1 froglets were subcloned and sequenced. Sequences highlighted in red and gray denote protospacer adjacent motif (PAM) and protospacer sequence, respectively. Splice donor site is underlined. The deleted and inserted nucleotides are shown by dashes and blue letters, respectively. Capital and small letters indicate exon and intron, respectively.

Based on our results from exogenous TH treatments, we expected that *trβ* crispants would fail to complete metamorphosis, or significantly extend the metamorphic period. To examine the effect of *trβ* disruption on natural metamorphosis, we observed morphological changes and measured the duration from fertilization to stage 61 immediately after gill resorption and prior to tail resorption, and to stage 66 when tail resorption is complete. There was no statistically significant difference in time from stage 1 to stage 61 between *trβ* crispants and control tadpoles from the same clutch (Fig. 5A). Unlike in TH-induced metamorphosis, gill resorption occurred normally in *trβ* crispants at the same time as in controls. However, we found significant differences in the length of the period from stage 61 to stage 66 (Fig. 5B,C) because tail resorption was slower in *trβ* crispants than in uninjected controls. Thus, *trβ* crispants took longer to accomplish metamorphosis completely. Metamorphosed *trβ* crispants started feeding normally, and the histological structure of the intestine at about one month after metamorphosis showed that intestinal remodeling was complete (Fig. S4). Interestingly, tentacles, which are specifically larval organs, persisted in most *trβ* crispants one week after metamorphosis but were resorbed completely within the next three weeks (Fig. S5). Importantly, we mated adult F0 crispants and confirmed that F1 offspring carrying compound heterozygous mutant alleles accomplished natural metamorphosis (Fig. 6).

## DISCUSSION

We have developed an efficient strategy to achieve mosaic knock-out founders (crispants) using sgRNA and Cas9 RNP in *X. tropicalis*. We demonstrated the effectiveness of this strategy by targeting an early exon and a splice junction in two melanin synthesis-related genes, *tyr* and *oca2*, with easily visible mutant phenotypes. Under optimized conditions, most injected embryos showed severe (full) albinism with low levels of mosaicism (<10%) and developmental defects (<15∼30%). Significantly, amplicon-sequencing analysis of the on-targets revealed that somatic mutation rates were high in severe *tyr* and *oca2* phenotypes, i.e. the wild-type allele reads were not, or rarely, detected. In the case of *tyr* sgRNA targeting exon 1, out-of-frame mutation rate was 89%, consistent with severe albinism in sequenced individuals. However, *oca2* sgRNA targeting the exon-intron junction induced indels adjacent to the splice donor site. By targeting splice site, mutant *oca*2 mRNA likely exhibited exon skipping or intron retention to lose its function (Radev et al., 2015). Weak and moderate phenotypes were hardly observed in *oca2* crispants compared to those of *tyr* potentially because splice targeting mutations have higher frequency of inducing null mutations compared to 66% when targeting coding regions. Combining out-of-frame and splicing site perturbation in *trβ* caused by co-injection of two sgRNA/Cas9 RNPs is expected to result in a high frequency of loss of protein function. Therefore, our sgRNA/Cas9 RNP-based strategy may rapidly facilitate the examination of the functions of genes of interest in this species.

We used this Cas9 RNP gene disruption strategy to generate *trβ* mosaic knock-out founders (‘*trβ* crispants’) and investigated the role of *trβ* in metamorphosis. A major phenotype in *trβ* crispants was delayed resorption of some larval tissues (tail and tentacles). The delay of tail resorption and tentacles in *trβ* crispants may be due to decreased ability to induce metamorphosis-related proteases. Indeed, the activation of two protease genes, *mmp13* and *fapa* by exogenous T3 was impaired in whole bodies of pre-metamorphic *trβ* crispants compared with wild-type tadpoles treated with T3. These proteases are well-known TH-response genes and are highly expressed during natural metamorphosis in sub-epidermal fibroblasts in the tail, notochord, notochord sheath, and gills, whose expression patterns are coincident with that of *trβ* (Brown et al., 1996; Berry et al., 1998a,b; Das et al., 2006). These results suggest that TRβ may be the main regulator of TH-induced activation of these protease genes and larval tissue resorption, at least for the tail.

In the gill, resorption was not observed in response to exogenous T3 in pre-metamorphic *trβ* crispants, even though it occurred normally in crispants during natural metamorphosis. It is unclear why loss of *trβ* showed different phenotypes of gills in T3-induced versus natural metamorphosis, though TRα expression levels may be sufficient to compensate for lack of TRβ at the climax of metamorphosis but not during pre-metamorphosis. Detailed analysis of the TH-regulated transcriptome, including expression of various proteases in all organs of *trβ* crispants during T3-induced and natural metamorphosis, is needed for further understanding.

Although the crispants were mosaic including in-frame mutations, *trβ* function is expected to be impaired at the organ and tissue level due to the 48-94% disruption of *trβ* by amplicon sequencing. Indeed, we observed natural metamorphosis in crispants, a phenotype confirmed by F1 bi-allelic mutants. Based on these results, we suggest that additional phenotypes observed in crispants, but not yet examined in non-mosaic *trβ* mutant animals, may accurately reflect the effect of loss of *trβ*. We observed no effects on limb development and intestinal remodeling in *trβ* crispants. Exogenous T3-induced precocious hind limb development occurred in *trβ* crispants as in wild-type controls. Also, the transcription factors, *klf9*, and *fra-2*, are highly expressed in developing and remodeling organs such as limb during natural metamorphosis (Wang and Brown, 1993; Berry et al., 1998b; Das et al., 2006), and normal induction by exogenous TH of these genes occurred in *trβ* crispants. These results are consistent with expectations on the small, if any, requirement for TRβ in the limb based on the high TRα to TRβ expression ratio in the limb and results from use of the TRα-selective antagonist CO23 (Wen et al., 2017; Ocasio and Scanlan, 2006).

At the molecular level, surprising results were obtained regarding the effects of TRβ on TH-response gene expression. Two of the best characterized TH direct response genes are *klf9* and *trβ* itself (Ranjan et al., 1994; Brown et al., 1996; Bagamasbad et al., 2008), but TH-induction of these genes gave contrasting results, namely only *trβ* and not *klf9* induction by exogenous TH was impaired in *trβ* crispants. Interestingly, in TRα knock-out tadpoles, induction by exogenous TH of both *klf9* and *trβ* was partially impaired (Choi et al., 2015; Wen and Shi, 2015). These results suggest that TRα is able to compensate for loss of TRβ with regard to *klf9* but not *trβ*. Possibilities to explain impaired *trβ* induction in *trβ* crispants are that (1) the two TRs exhibit isoform specificity in molecular mechanisms in regulation of *trβ*, (2) tissue-specific expression of the TR isoforms exists such that *trβ* and little *trα* is expressed in tissues that exhibit *trβ* autoregulation (e.g. red blood cells and brain subventricular cells) (Denver et al., 2009), or (3) *trβ* mRNA level is decreased by NMD. Similar possible explanations apply to the other TH response genes studied, *fra-2*, *mmp13*, and *fapa*. Further research is required to examine these intriguing possibilities. Although we only analyzed the perturbation of TH-response gene expression using total RNA from whole bodies in this study, various organ and tissue actually showed less responsiveness to TH in the *trβ* crispants; delay of gill, tail and tentacle resorption during T3-induced and natural metamorphosis. To understand the differences of responsiveness to TH mediated by *trβ* in each organ, we need to further analyze transcriptome changes in each organ.

Another surprising result from *trβ* crispants was that they developed normally and were able to complete metamorphosis through tail resorption, albeit with a delay. This result suggests that TRβ is not required for metamorphosis but plays a role in timing of developmental events already fated to occur as specified during development. At least three hypotheses explain why *trβ* crispants metamorphosed completely. First, TRα may partially compensate for TRβ deficiency so that the metamorphosis program could occur. Second, rather than TH-response gene induction by TRα, derepressed levels (i.e. above basal levels but below TH-induced levels) of TH response gene expression due to lack of TRβ-mediated repression, as detailed by the dual function model (Shi, 2009), may explain the ability to progress through tail resorption. These two hypotheses also explain the delay in metamorphosis in *trβ* crispants where reduced gene expression levels below that of full induction as occurs in wild-type tadpoles would increase the time required to progress through metamorphosis. Third, unknown mechanisms related to TH carried out metamorphosis (e.g. non-genomic pathway of TH). To address this question, we need to produce and analyze *trα*/*trβ* double knock-out frogs in the future.

In conclusion, we show that, despite dramatic contrasting effects on TH response gene expression observed in *trβ* crispants, TRβ has significant but mild effects on developmental timing that do not prevent complete metamorphosis from occurring. These results, in combination with results from TRα knock-out animals, provide evidence that TRα and TRβ may at least partly functionally compensate for each other but that TRα and TRβ have separate functions during metamorphosis*.* Full evaluation of the role of TRs in amphibian development await analysis of TRα and TRβ double knock-out animals.

## MATERIALS AND METHODS

### Animal rearing and treatment

*Xenopus tropicalis*, the Golden strain, were provided by the Amphibian Research Center (Hiroshima University) through the National Bio-Resource Project of the AMED, Japan. Eggs and testes were collected from sexually mature adult frogs with an injection of human chorionic gonadotropin (Aska Pharmaceutical, Tokyo, Japan), and *in vitro* fertilization was carried out. To isolate testes, male frogs were treated with 1% MS-222 (tricaine; Sigma-Aldrich, MO, USA) as anesthesia and euthanasia. One-cell-stage embryos were de-jellied with 2% L-cysteine solution. After washing with 0.1×Marc's modified ringer (MMR), embryos were microinjected in 6% Ficoll (Sigma-Aldrich) in 0.33×MMR. At the blastula stage, embryos were moved to 0.1×MMR. At 16–20 h after fertilization, normally developed embryos were collected and counted and represented the initial numbers of individuals in each experiment. Embryos and tadpoles were reared at 25–26°C. Tadpoles at stage 52–54 were treated with 10 nM 3,3,5-triiodo-L-thyronine (T3; Sigma-Aldrich) for 3 days. Animal rearing and treatments were performed and approved according to the Hiroshima University guidelines for the use and care of experimental animals.

### Preparation and microinjection of sgRNA/Cas9 ribonucleoprotein

All sgRNA targeting sequences and oligonucleotide information are listed in Tables S1 and S2. The sgRNA targeting sequence of *tyrosinase* (*tyr*) was taken from Blitz et al. (2013), Nakayama et al. (2013). *oculocutaneous albinism 2* (*oca2*) and *thyroid hormone receptor beta* (*trβ*) sgRNAs were designed using CRISPR-direct (Naito et al., 2015). *tyr* and one of the *trβ* sgRNAs were designed within the exon, whereas *oca2* and the other *trβ* sgRNA was designed across the exon-intron junction to induce splicing errors (Radev et al., 2015). The annealed oligonucleotides for *tyr* were cloned into the *BsmBI* site of the pCS2P-gRNA vector, and subsequently amplified for *in vitro* transcription templates using KOD FX Neo (TOYOBO, Osaka, Japan) and primer sets (IVT-sgRNA-F and R), as described previously (Ota et al., 2014; Shigeta et al., 2016). The other sgRNA templates were assembled by a PCR-based strategy (Nakayama et al., 2014; Sakane et al., 2017). DNA templates were purified with a QIAquick PCR Purification Kit (Qiagen, Hilden, Germany), subsequently, sgRNAs were synthesized using a MEGAshortscript T7 Kit (Thermo Fisher Scientific, MA, USA) and purified using a MEGAclear Kit (Thermo Fisher Scientific). Before microinjection, recombinant Cas9 protein (Alt-R *S.p*. Cas9 Nuclease 3NLS; Integrated DNA Technologies, IA, USA) and sgRNA(s) were incubated in 150 mM KCl and 20 mM HEPES buffer to form ribonucleoprotein complexes at room temperature for 10 min. Mixtures of Cas9 1 ng protein and 200 pg of one of the two sgRNAs were injected into one-cell-stage *X. tropicalis* embryos using a Nanoject II (Drummond, PA, USA).

### Preparation of genomic DNA

Uninjected control embryos and *tyr-*, *oca2-*, and *trβ-*sgRNA/Cas9 RNP-injected embryos were individually collected at after 1 day or 3 days of injection. *trβ* crispants and F1 offspring were individually collected at stage 66. Each genomic DNA was extracted from the whole bodies or the tips of digits using a DNeasy Blood and Tissue Kit (Qiagen).

### RNA extraction, RT-PCR, and quantitative PCR

For RT-PCR, individual tadpoles were collected at stage 61/62 (*n*=6). For quantitative PCR, four or more tadpoles at stage 52-54 were pooled in each sample for each treatment with and without T3 (*n*=3), and this experiment was performed three times independently. Tadpoles were homogenized in liquid nitrogen. Total RNA was extracted by RNAiso (TaKaRa, Shiga, Japan) and purified with Direct-zol™ RNA MiniPrep (Zymo Research, CA, USA). The same quantity of total RNA was reverse transcribed with ReverTra Ace qPCR RT Master Mix with a gDNA Remover kit (TOYOBO, Osaka, Japan). RT-PCR was performed using KOD FX Neo (TOYOBO) and analyzed using a microchip electrophoresis system (DNA-500 reagent kit and MCE-202 MultiNA; Shimadzu, Kyoto, Japan). Quantitative PCR (qPCR) was carried out by using KOD SYBR qPCR Mix (TOYOBO) with a Step One real-time PCR system (Thermo Fisher Scientific). Three technical replicates were used per sample and three biological replicates were analyzed in each experiment. Target gene expression levels were normalized by the expression of the housekeeping gene *rpl8*. RT-PCR and qPCR primer sets are listed in Table S2. All primer sets were designed by Primer 3 (Untergasser et al., 2012).

### HMA and amplicon sequencing

For HMA, PCR on genomic DNA was performed over 35 cycles using KOD FX Neo (TOYOBO), and PCR products were analyzed using a microchip electrophoresis system (DNA-500 reagent kit and MCE-202 MultiNA; Shimadzu) according to Shigeta et al. (2016). An amplicon-sequencing library was prepared based on the Illumina ‘16S Metagenomic Sequencing Library Preparation’. For the first round of PCR, the target regions containing sgRNA targeting sites were amplified from individual genomic DNA of uninjected control embryos (*n*=5), *tyr* (*n*=10), *oca2* (*n*=5), and *trβ* (*n*=8) crispants using a KAPA HiFi HS ReadyMix (NIPPON Genetics, Tokyo, Japan) with primer sets containing barcode and overhang adaptor sequences. Equal quantities of all PCR products were pooled and purified using a QIAquick PCR Purification Kit (Qiagen). The second round of PCR was performed to construct a sequence library using a Nextra XT index kit (Illumina, CA, USA). The final library was purified and sequenced on the Illumina MiSeq. Library construction and sequencing were performed by Macrogen Japan and Hokkaido System Science. All primers are listed in Table S2.

### Amplicon data analysis

Sequence data were preprocessed by Scythe (v0.994) (https://github.com/vsbuffalo/scythe) and Sickle programs (Joshi and Fass, 2011; https://github.com/najoshi/sickle) to trim adaptor sequences and remove reads that were shorter than 36 bp. Then, trimmed paired-end reads were joined into single reads using fastq-join (version 1.1.2-537) (Aronesty, 2011; https://github.com/ExpressionAnalysis/ea-utils). PCR and Illumina sequence error rates were accounted for using wild-type samples, and then wild-type reads and mutant reads for *tyr*, *oca2*, and *trβ* were counted using an in-house script in R (version 3.3.3).

### Histological analysis

*trβ* crispants were genotyped by HMA using genomic DNA from the tips of digits. Whole body and intestine at about one month after metamorphosis were fixed in 95% ethanol. Isolated intestines were embedded in paraffin, sectioned at 5 µm and stained with hematoxylin and eosin (Choi et al., 2017).

### Statistical analysis

Welch's *t*-test was used for qPCR analysis and duration from fertilization to adult. When the *P*-value is less than 0.05, we conclude that a significant difference exists.

## Supplementary Material

Supplementary information
