# Supplementary material for: Functional analysis of thyroid hormone receptor beta in Xenopus tropicalis founders using CRISPR-Cas
Source: Biol Open. 2018 Jan 15;7(1):bio030338. doi: 10.1242/bio.030338 (PMC5829506; doi:10.1242/bio.030338)
Supplement: Supplementary information [file biolopen-7-030338-s1.pdf]

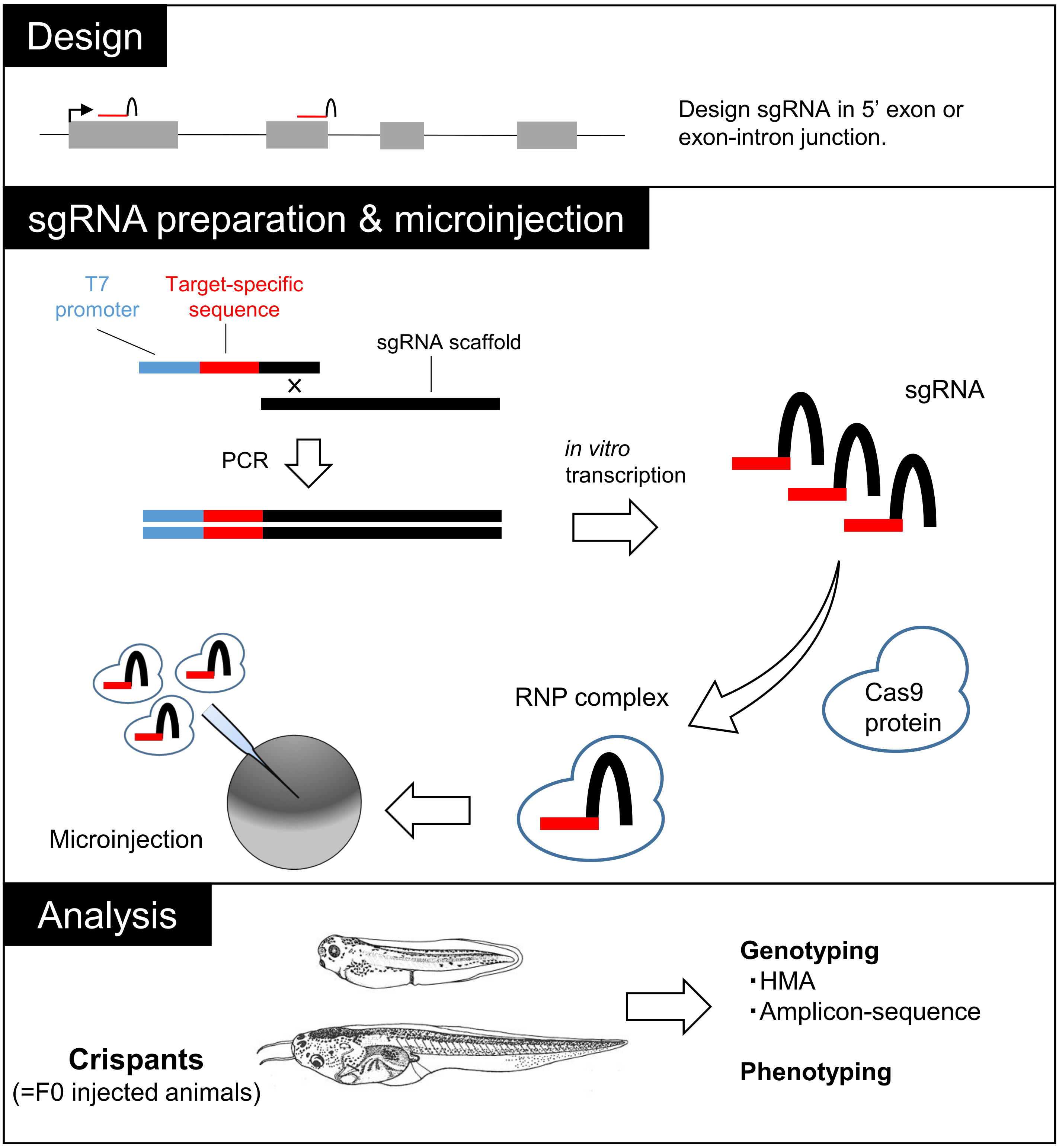

Biology Open • Supplementary information

Fig. S1: A workflow for sgRNA/Cas9 RNP injection into *Xenopus tropicalis*

We established an efficient and rapid gene disruption workflow for the loss-of-function analysis using the CRISPR-Cas system in *X. tropicalis* founders. First, we design one or more sgRNAs targeting 5’ exons or exon-intron junctions to induce frameshift mutations and/or splicing errors. Next, sgRNAs are synthesized *in vitro* by T7 RNA polymerase using templates produced by PCR and assembled with Cas9 protein to form an RNP complex. sgRNA/Cas9 RNP is injected into one-cell-stage fertilized eggs, and the developing embryos (crispants) are genotyped by HMA and amplicon-sequencing followed by phenotyping.

A

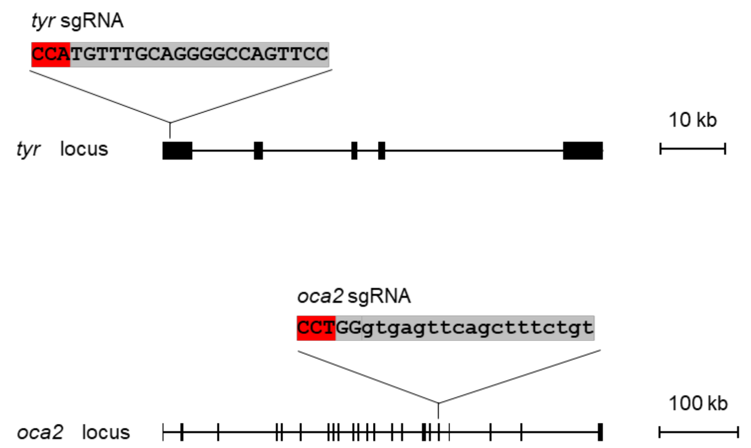

B

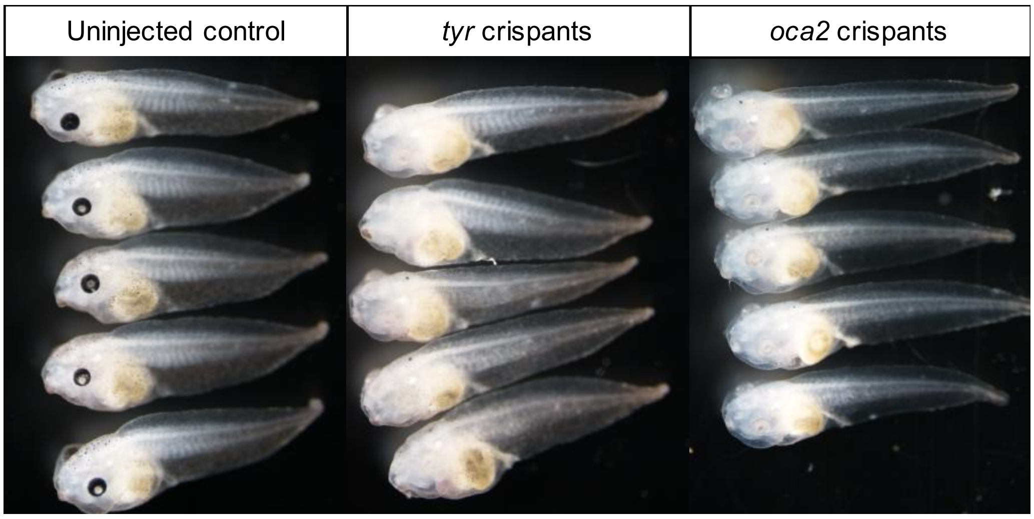

C

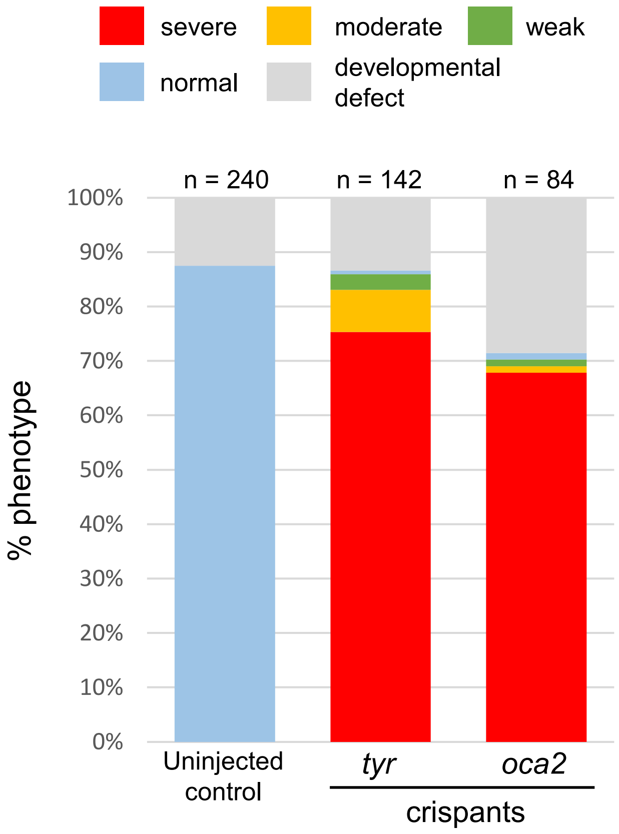

D

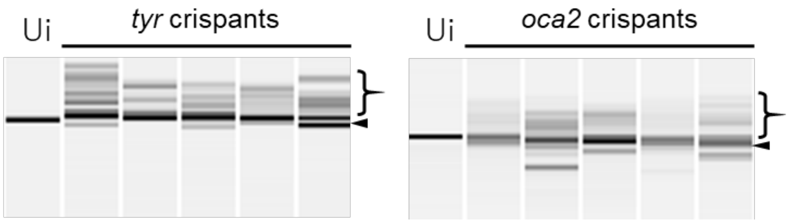

E

target gene (total crispant samples)	*tyr* (n = 10)	*oca2* (n = 5)
wild-type reads (%)	0 (0 %)	0 (0 %)
mutant reads (%)	24936 (100 %)	13309 (100 %)
total reads	24936	13309

Fig. S2: Phenotyping and genotyping of embryos with disrupted melanin synthesis-related genes

(A) Schematic illustration of the gene structure and sgRNA targeting sequences of *tyrosinase* (*tyr*) and *oculocutaneous albinism 2* (*oca2*) genes. Coding regions are shown as black boxes. Sequences highlighted in red and gray denote protospacer adjacent motif (PAM) and the 20 bp target sequence of sgRNA, respectively. Capital letters: exon; small letters: intron. (B) Representative severe phenotypes of *tyr* and *oca2* crispants. (C) Phenotypes were classified into four groups: severe, near complete loss of pigmentation in retinal pigmented epithelium; moderate, more than half of pigmentation lost; weak, less than half of pigmentation lost; and normal, no alteration in pigmentation (Shigeta et al., 2016; Sakane et al., 2017). (D) Genotyping of crispants with HMA. Target regions were amplified with 35 cycles of PCR. Genomic PCR products were analyzed using microchip electrophoresis. Arrowheads and brackets indicate homoduplex and heteroduplex bands, respectively. Ui: uninjected control embryos. (E) The numbers and percentages of wild-type and mutant allele reads in each crispant. Reads obtained from next-generation sequencing were classified into each gene and individual by their specific barcode sequences. According to sequence data from uninjected control embryos, the rates of artificial backgrounds such as PCR or sequencing errors were calculated, and we discounted reads below these artificial background rates. Significant reads in ten *tyr* and five *oca2* crispants were counted as total reads and somatic mutation rates were calculated.

Biology Open • Supplementary information

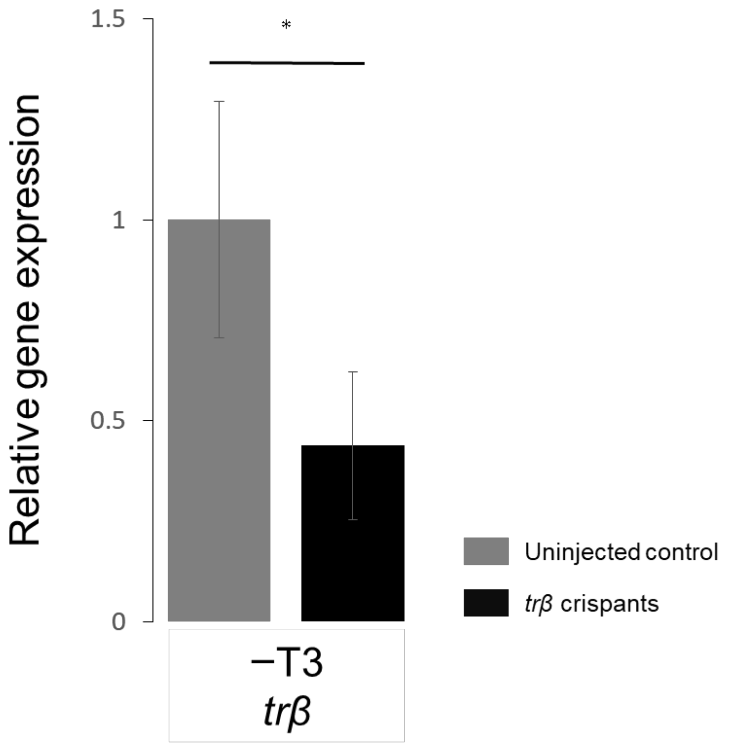

Fig. S3: mRNA expression of *trβ* in the absent of T3

The result of *trβ* expression in control and crispants without T3. The scale of Y axis is enlarged in Fig. 4. *, $P < 0.005$; Welch's *t*-test.

Biology Open • Supplementary information

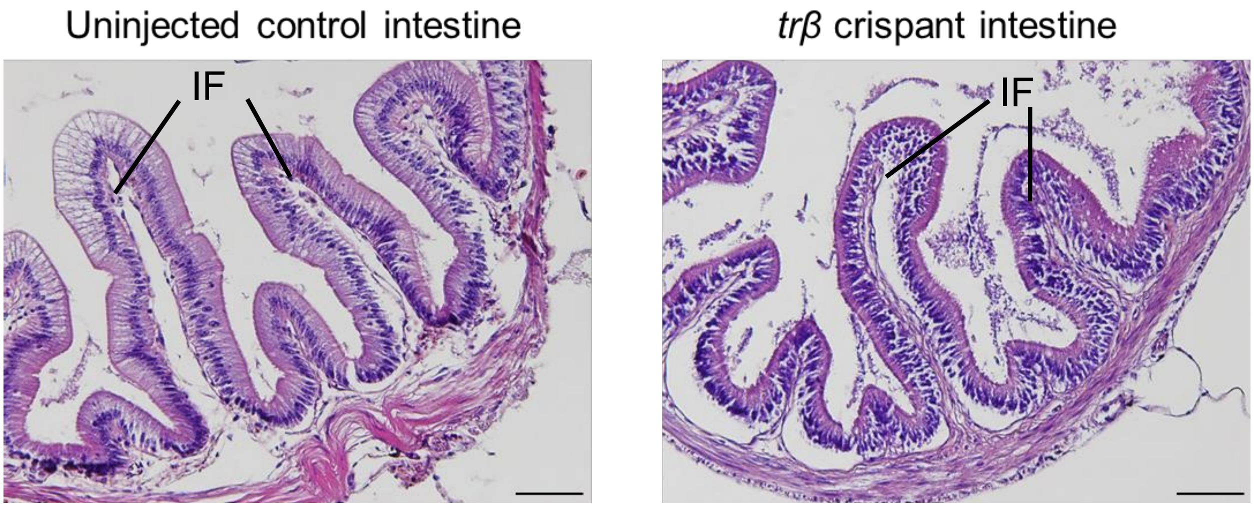

Fig. S4: Histological analysis of intestine

Intestines of uninjected control froglets and *trβ* crispants about one month after metamorphosis were processed for histology and stained with hematoxylin and eosin. Intestinal folds (IF), which develop only in the adult organ, were observed in both control froglets and crispants. Bar: 50 μ m.

Biology Open • Supplementary information

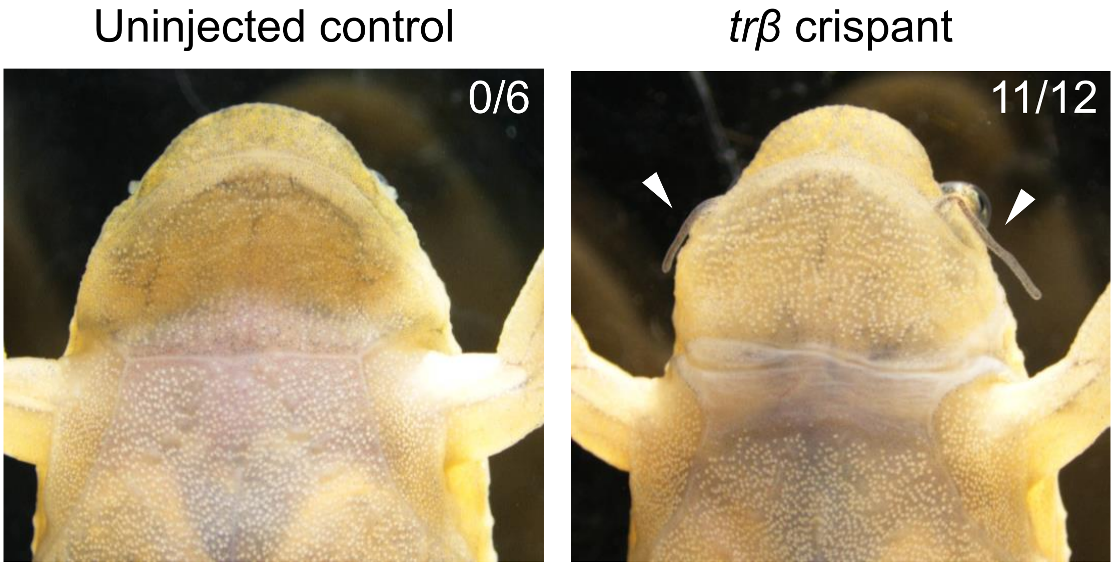

Fig. S5: Tentacle resorption was delayed in *trβ* crispants

About one week after metamorphosis, tentacles remained near the mouth of *trβ* crispants (arrowheads). Images are representative of six uninjected control froglets and eleven *trβ* crispants. The number of froglets with remaining tentacle(s) is given in the images.

Biology Open • Supplementary information

Supplementary information table

Table S1: Sequences of sgRNA targeting sites.

sgRNA name	Target sites (5' to 3')
tyr	CCATGTTTGCAGGGGCCAGTTCC
oca2	CCTGGGTGAGTTCAGCTTTCTGT
trβ-1	GATGAGCTATGCGTGGTGTGTGG
trβ-2	AGGGCTGCAAGGTAACAGATTGG

PAM sequence is underlined.

Table S2: Oligonucleotide information in this study.

Primers used for HMA.

Target gene	Forward primer sequence (5' to 3')	Reverse primer sequence (5' to 3')
tyr	AAGCCTGCACATGTGATTGTGCTTG	GAGGACCCATCTCCAGACCACACAG
oca2	TGTTTGTCTGTCTTGGGCTTTGTCA	GGGGTTACAGCACTGATATGACTATGG
trβ	TTTCTTTCTTTGCTTTCCTTTATCA	ACTGCTGTAGCGCAGGGTTC

Oligonucleotides used for PCR assembly for sgRNA template.

sgRNA name	Forward oligonucleotide sequence (5' to 3')
oca2	TAATACGACTCACTATAGGAGAAAGCTGAACTCACCCGTTTTAGAGCTAGAAATAGCAAG
trβ-1	TAATACGACTCACTATAGGTGAGCTATGCGTGGTGTGGTTTTAGAGCTAGAAATAGCAAG
trβ-2	TAATACGACTCACTATAGGGGCTGCAAGGTAACAGATGTTTTAGAGCTAGAAATAGCAAG

Reverse oligonucleotide sequence (5' to 3')	AAAAGCACCGACTCGGTGCCACTTTTTCAAGTTGATAACGGACTAGCCTTATTTTAACTTGCTATTTCTAGCTCTAAAAC

Biology Open • Supplementary information

Oligonucleotides used for constructing sgRNA vector.

sgRNA name	Sequence (5' to 3')	
tyr	Sense	TATAGGAACTGGCCCCTGCAAACAG
	Antisense	AAAACTGTTTGCAGGGGCCAGTTCC

Primers for sgRNA *in vitro* transcription template.

Primer name	Sequence (5' to 3')
IVT-gRNA-F	GAATTCTAATACGACTCAC
IVT-gRNA-R	AAAAGCACCGACTCGG

Primers for the amplicon sequence.

The overhang adapter and barcode sequences are added to the locus-specific primer. Barcode sequences are indicated by small letters.

Primer name	Sequence (5' to 3')
tyr-Wt-ampliconseq1-F	TCGTCGGCAGCGTCAGATGTGTATAAGAGACAGttaaAGCAGCATGGAAAGGAACATG
tyr-Wt-ampliconseq1-R	GTCTCGTGGGCTCGGAGATGTGTATAAGAGACAGttaaCAGAGCTGGTCAGGACAACATC
tyr-Wt-ampliconseq2-F	TCGTCGGCAGCGTCAGATGTGTATAAGAGACAGaacgAGCAGCATGGAAAGGAACATG
tyr-Wt-ampliconseq2-R	GTCTCGTGGGCTCGGAGATGTGTATAAGAGACAGatcgCAGAGCTGGTCAGGACAACATC
tyr-Wt-ampliconseq3-F	TCGTCGGCAGCGTCAGATGTGTATAAGAGACAGagcgAGCAGCATGGAAAGGAACATG
tyr-Wt-ampliconseq3-R	GTCTCGTGGGCTCGGAGATGTGTATAAGAGACAGaccgCAGAGCTGGTCAGGACAACATC
tyr-Wt-ampliconseq4-F	TCGTCGGCAGCGTCAGATGTGTATAAGAGACAGacgAGCAGCATGGAAAGGAACATG
tyr-Wt-ampliconseq4-R	GTCTCGTGGGCTCGGAGATGTGTATAAGAGACAGgtagCAGAGCTGGTCAGGACAACATC

Biology Open • Supplementary information

tyr-Wt-ampliconseq5-F	TCGTCGGCAGCGTCAGATGTGTATAAGAGACAGcacgAGCAGCATGGAAAGGAACATG
tyr-Wt-ampliconseq5-R	GTCTCGTGGGCTCGGAGATGTGTATAAGAGACAGtatgCAGAGCTGGTCAGGACAACATC
oca2-Wt-ampliconseq1-F	TCGTCGGCAGCGTCAGATGTGTATAAGAGACAGcaatATGTTTGTCTGTCTTGGGCTTT
oca2-Wt-ampliconseq1-R	GTCTCGTGGGCTCGGAGATGTGTATAAGAGACAGctatAAGGGGTTACAGCACTGATATG
oca2-Wt-ampliconseq2-F	TCGTCGGCAGCGTCAGATGTGTATAAGAGACAGacacATGTTTGTCTGTCTTGGGCTTT
oca2-Wt-ampliconseq2-R	GTCTCGTGGGCTCGGAGATGTGTATAAGAGACAGgactAAGGGGTTACAGCACTGATATG
oca2-Wt-ampliconseq3-F	TCGTCGGCAGCGTCAGATGTGTATAAGAGACAGttacATGTTTGTCTGTCTTGGGCTTT
oca2-Wt-ampliconseq3-R	GTCTCGTGGGCTCGGAGATGTGTATAAGAGACAGtgacAAGGGGTTACAGCACTGATATG
oca2-Wt-ampliconseq4-F	TCGTCGGCAGCGTCAGATGTGTATAAGAGACAGaatgATGTTTGTCTGTCTTGGGCTTT
oca2-Wt-ampliconseq4-R	GTCTCGTGGGCTCGGAGATGTGTATAAGAGACAGtgcaAAGGGGTTACAGCACTGATATG
oca2-Wt-ampliconseq5-F	TCGTCGGCAGCGTCAGATGTGTATAAGAGACAGgtacATGTTTGTCTGTCTTGGGCTTT
oca2-Wt-ampliconseq5-R	GTCTCGTGGGCTCGGAGATGTGTATAAGAGACAGggacAAGGGGTTACAGCACTGATATG

Primer name	Sequence (5’ to 3’)
tyr-Mt-ampliconseq1-F	TCGTCGGCAGCGTCAGATGTGTATAAGAGACAGttaaAGCAGCATGGAAAGGAACATG
tyr-Mt-ampliconseq1-R	GTCTCGTGGGCTCGGAGATGTGTATAAGAGACAGttaaCAGAGCTGGTCAGGACAACATC
tyr-Mt-ampliconseq2-F	TCGTCGGCAGCGTCAGATGTGTATAAGAGACAGaacgAGCAGCATGGAAAGGAACATG

Biology Open • Supplementary information

tyr-Mt-ampliconseq2-R	GTCTCGTGGGCTCGGAGATGTGTATAAGAGACAGatcgCAGAGCTGGTCAGGACAACATC
tyr-Mt-ampliconseq3-F	TCGTCGGCAGCGTCAGATGTGTATAAGAGACAGagcgAGCAGCATGGAAAGGAACATG
tyr-Mt-ampliconseq3-R	GTCTCGTGGGCTCGGAGATGTGTATAAGAGACAGaccgCAGAGCTGGTCAGGACAACATC
tyr-Mt-ampliconseq4-F	TCGTCGGCAGCGTCAGATGTGTATAAGAGACAGacgAGCAGCATGGAAAGGAACATG
tyr-Mt-ampliconseq4-R	GTCTCGTGGGCTCGGAGATGTGTATAAGAGACAGgtagCAGAGCTGGTCAGGACAACATC
tyr-Mt-ampliconseq5-F	TCGTCGGCAGCGTCAGATGTGTATAAGAGACAGcacgAGCAGCATGGAAAGGAACATG
tyr-Mt-ampliconseq5-R	GTCTCGTGGGCTCGGAGATGTGTATAAGAGACAGtatgCAGAGCTGGTCAGGACAACATC
tyr-Mt-ampliconseq6-F	TCGTCGGCAGCGTCAGATGTGTATAAGAGACAGttcgAGCAGCATGGAAAGGAACATG
tyr-Mt-ampliconseq6-R	GTCTCGTGGGCTCGGAGATGTGTATAAGAGACAGtccgCAGAGCTGGTCAGGACAACATC
tyr-Mt-ampliconseq7-F	TCGTCGGCAGCGTCAGATGTGTATAAGAGACAGtgcgAGCAGCATGGAAAGGAACATG
tyr-Mt-ampliconseq7-R	GTCTCGTGGGCTCGGAGATGTGTATAAGAGACAGggttCAGAGCTGGTCAGGACAACATC
tyr-Mt-ampliconseq8-F	TCGTCGGCAGCGTCAGATGTGTATAAGAGACAGctcgAGCAGCATGGAAAGGAACATG
tyr-Mt-ampliconseq8-R	GTCTCGTGGGCTCGGAGATGTGTATAAGAGACAGcccgCAGAGCTGGTCAGGACAACATC
tyr-Mt-ampliconseq9-F	TCGTCGGCAGCGTCAGATGTGTATAAGAGACAGcgcgAGCAGCATGGAAAGGAACATG
tyr-Mt-ampliconseq9-R	GTCTCGTGGGCTCGGAGATGTGTATAAGAGACAGgacgCAGAGCTGGTCAGGACAACATC
tyr-Mt-ampliconseq10-F	TCGTCGGCAGCGTCAGATGTGTATAAGAGACAGgtcgAGCAGCATGGAAAGGAACATG

Biology Open • Supplementary information

tyr-Mt-ampliconseq10-R	GTCTCGTGGGCTCGGAGATGTGTATAAGAGACAGgccgCAGAGCTGGTCAGGACAACATC
oca2-Mt-ampliconseq1-F	TCGTCGGCAGCGTCAGATGTGTATAAGAGACAGcaatATGTTTGTCTGTCTTGGGCTTT
oca2-Mt-ampliconseq1-R	GTCTCGTGGGCTCGGAGATGTGTATAAGAGACAGctatAAGGGGTTACAGCACTGATATG
oca2-Mt-ampliconseq2-F	TCGTCGGCAGCGTCAGATGTGTATAAGAGACAGacacATGTTTGTCTGTCTTGGGCTTT
oca2-Mt-ampliconseq2-R	GTCTCGTGGGCTCGGAGATGTGTATAAGAGACAGgactAAGGGGTTACAGCACTGATATG
oca2-Mt-ampliconseq3-F	TCGTCGGCAGCGTCAGATGTGTATAAGAGACAGttacATGTTTGTCTGTCTTGGGCTTT
oca2-Mt-ampliconseq3-R	GTCTCGTGGGCTCGGAGATGTGTATAAGAGACAGtgacAAGGGGTTACAGCACTGATATG
oca2-Mt-ampliconseq4-F	TCGTCGGCAGCGTCAGATGTGTATAAGAGACAGaatgATGTTTGTCTGTCTTGGGCTTT
oca2-Mt-ampliconseq4-R	GTCTCGTGGGCTCGGAGATGTGTATAAGAGACAGtgcaAAGGGGTTACAGCACTGATATG
oca2-Mt-ampliconseq5-F	TCGTCGGCAGCGTCAGATGTGTATAAGAGACAGgtacATGTTTGTCTGTCTTGGGCTTT
oca2-Mt-ampliconseq5-R	GTCTCGTGGGCTCGGAGATGTGTATAAGAGACAGggacAAGGGGTTACAGCACTGATATG

Primer name	Sequence (5' to 3')
trβ-Mt-ampliconseq1-F	TCGTCGGCAGCGTCAGATGTGTATAAGAGACAGacgGGTATCAAAGGGTGTCCAAAAA
trβ-Mt-ampliconseq1-R	GTCTCGTGGGCTCGGAGATGTGTATAAGAGACAGgtagATTTCCAGGCAGTGCCAATA
trβ-Mt-ampliconseq2-F	TCGTCGGCAGCGTCAGATGTGTATAAGAGACAGcacgGGTATCAAAGGGTGTCCAAAAA
trβ-Mt-ampliconseq2-R	GTCTCGTGGGCTCGGAGATGTGTATAAGAGACAGtatgATTTCCAGGCAGTGCCAATA

Biology Open • Supplementary information

trβ-Mt-ampliconseq3-F	TCGTCGGCAGCGTCAGATGTGTATAAGAGACAGttcgGGTATCAAAGGGTGTCCAAAAA
trβ-Mt-ampliconseq3-R	GTCTCGTGGGCTCGGAGATGTGTATAAGAGACAGtccgATTTCCAGGCAGTGCCAATA
trβ-Mt-ampliconseq4-F	TCGTCGGCAGCGTCAGATGTGTATAAGAGACAGtgcgGGTATCAAAGGGTGTCCAAAAA
trβ-Mt-ampliconseq4-R	GTCTCGTGGGCTCGGAGATGTGTATAAGAGACAGggttATTTCCAGGCAGTGCCAATA
trβ-Mt-ampliconseq5-F	TCGTCGGCAGCGTCAGATGTGTATAAGAGACAGctcgGGTATCAAAGGGTGTCCAAAAA
trβ-Mt-ampliconseq5-R	GTCTCGTGGGCTCGGAGATGTGTATAAGAGACAGcccgATTTCCAGGCAGTGCCAATA
trβ-Mt-ampliconseq6-F	TCGTCGGCAGCGTCAGATGTGTATAAGAGACAGcgcgGGTATCAAAGGGTGTCCAAAAA
trβ-Mt-ampliconseq6-R	GTCTCGTGGGCTCGGAGATGTGTATAAGAGACAGgacgATTTCCAGGCAGTGCCAATA
trβ-Mt-ampliconseq7-F	TCGTCGGCAGCGTCAGATGTGTATAAGAGACAGgtcgGGTATCAAAGGGTGTCCAAAAA
trβ-Mt-ampliconseq7-R	GTCTCGTGGGCTCGGAGATGTGTATAAGAGACAGgccgATTTCCAGGCAGTGCCAATA
trβ-Mt-ampliconseq8-F	TCGTCGGCAGCGTCAGATGTGTATAAGAGACAGcaatGGTATCAAAGGGTGTCCAAAAA
trβ-Mt-ampliconseq8-R	GTCTCGTGGGCTCGGAGATGTGTATAAGAGACAGctatATTTCCAGGCAGTGCCAATA

Primers used for RT-PCR.

Target gene	Forward primer sequence (5' to 3')	Reverse primer sequence (5' to 3')
trβ	AAGAAACAAAACCTGGCCATC	AAGCGACATTCTTGGCACT

Primers used for qPCR

Target gene	Forward primer sequence (5' to 3')	Reverse primer sequence (5' to 3')

Biology Open • Supplementary information

rpl8	CCCTCAACCATCAGGAGAGA	TCTTTGTACCACGCAGACGA
trβ	ATAATGCCAAGCAGTATGTCAGAGA	CACACACCACGCATAGCTCAT
klf9	GGTGTCCTTATGCTGGGTGT	GCGAGTCAACTCATCGGAAC
fra-2	TCCCATCCTTACAGCCACTC	TTCTCCTGCTCCAGCTTCTC
mmp13	CCGAGGGTTACTTGGACAAA	CACCGACATCCCAAAGAACT
fapa	ATCCTGGGGAAAAGCCTCTA	CTGGGGCCTCTTCATATTCA

Biology Open • Supplementary information
